# Active Tuberculosis in Neighboring Households of an Index Case in Mwenga Health Zone, South Kivu, DR Congo July—August 2021

**DOI:** 10.1093/ofid/ofag725

**Published:** 2026-11-13

**Authors:** Eric Mulume Musafiri, Christian Chirhibagula, Didier Muhima, Freddy Birembano, Jean Stephane Mbuyi, Jean Pierre Tshibuyi Malemba, Esto Bahizire

**Affiliations:** National TB Program of the DRC, Provincial Office of South Kivu, Laboratory, Bukavu, DRC; Univesité Catholique de Graben Butembo, Faculté de Médecine, Butembo, RDC; Institut Supérieur de Techniques Médicales de Kaziba, Bukavu, DRC; Ecole Régionale de Santé Publique de l’UCB Bukavu, Pool de recherche en santé, Bukavu, RDC; The Leprosy Mission (TLM) Bureau de Bukavu, Suivi et évaluation, Bukavu, RDC; National TB Program of the DRC, Provincial Office of South Kivu, Laboratory, Bukavu, DRC; National TB Program of the DRC, National Office of Kinshasa, Kinshasa, RDC; National TB Program of the DRC, National Office of Kinshasa, Kinshasa, RDC; Center for Tropical Diseases & Global Health, Catholic University of Bukavu, Bukavu, DRC; Centre de Recherche en Sciences Naturelles (CRSN) de Lwiro, departement de la tuberculose, Bukavu, DRC; Department of Medical Microbiology, University of Nairobi, Nairobi, Kenya

**Keywords:** index case, neighboring, TB

## Abstract

**Background:**

The Democratic Republic of the Congo (DRC) is among the 10 high-burden tuberculosis (TB) countries, with an estimated detection gap of 70%. This study aimed to assess the prevalence of TB in index case households and neighboring households in Mwenga, a rural Health Zone (HZ), Est Democratic Republic of the Congo.

**Method:**

We conducted a cross-sectional study to screen for active TB by microscopy and thereafter confirmed by Xpert MTB/RIF among household and neighbors of 187 index cases identified from TB registries in South Kivu Mwenga HZ. Awareness campaigns and screenings were conducted for all members of the index case households and their neighboring households. Individuals with TB suggestive symptoms who consented were interviewed and tested. R software was used to assess association between TB status and risk factors in both household types.

**Results:**

Prevalence of active TB in the surveyed households was 250/1707 (14.7%) of pulmonary and extrapulmonary TB, with 219 (12.8%) bacteriologically confirmed. Similar risk was observed between index households (65/493 [13.2%] TB-positive) and neighboring households (154/1214 [12.7%] TB-positive, *P* = .84). Factors significantly associated with TB positivity included cough (adjusted odds ratio [AOR] 1.92, 95% confidence interval [CI]: 1.21–3.11, *P* = .006), age >65 years (AOR 2.13, 95% CI: 1.34–3.39, *P* = .001), and night sweats (AOR 1.50, 95% CI: 1.12–2.00, *P* = .006).

**Conclusions:**

The study highlights a similar risk of contracting TB in neighboring households compared to index case households. Efforts should focus on contact tracing in both household types. These findings have implications for TB control programs in the DRC and other high-prevalence countries.

The world health organization (WHO) End TB Strategy aims to reduce the tuberculosis (TB) incidence by 90% and TB-related deaths by 95% by 2035 [1]. To address the global TB epidemic, the first United Nations High-Level Meeting on TB in 2018 set a target to close the detection gap by 2022 [2]. However, countries like the Democratic Republic of the Congo (DRC) face a 70% detection gap, highlighting the need for intensified efforts [3]. Dr. Matshidiso Moeti, WHO Regional Director for Africa, emphasized the urgency of scaling up responses to mitigate the suffering and deaths caused by TB [4].

Globally, TB remains a major cause of morbidity and mortality, driven by poverty, malnutrition, overcrowding, and immunosuppression [3, 5, 6]. In 2019, TB caused 1.3 million deaths, making it the deadliest infectious disease worldwide [7]. Challenges in delivering and accessing essential TB control services have left many cases undiagnosed globally [8]. In 2021, the DRC reported a contact investigation rate of only 8.1%, with South Kivu Province reporting an even lower rate of 6.5% [9]. Mwenga Health Zone (HZ) lagged further behind at 3.8%, with a TB incidence of 220 per 100 000 inhabitants [10], higher than the average in South Kivu (131/100 000 inhabitants) [9].

From 213 index cases, 514 household and 4141 neighboring contacts underwent TB screening: 19 household (3.70%, 95% confidence interval [CI] 2.24–5.71) and 45 neighboring (1.09%, 95% CI: .79–1.45) contacts were identified with TB, of whom 18 (3.50%, 95% CI: 2.20–5.48) and 38 (.92%, 95% CI: .65–1.13), respectively, were bacteriologically confirmed [11].

These contacts are analyzed on the basis of proximity and interaction frequency, in accordance with the concentric circles model. The risk correlates directly with closer and more frequent contact: primary contacts comprise the closest and most regular associations, such as family members, colleagues, and classmates, characterized by daily interaction; secondary contacts involve frequent interactions occurring several times a week within enclosed environments, including sports clubs and recreational venues; tertiary contacts consist of more occasional interactions, taking place only a few times per month [12].

Current TB contact investigation practices in the DRC focus on household and rarely on workplace contacts, neglecting neighboring households that may also be at risk. Studies on TB prevalence in neighboring households are limited, yet some data suggest they may face similar risks as household members [13].

The proposed study aimed to expand the scope of TB contact investigation to include neighboring households, hypothesizing that individuals in these households are at equal risk of developing TB. By focusing on Mwenga HZ, this study sought to determine TB prevalence in index and neighboring households and identify associated risk factors.

The findings are intended to inform strategies for reducing the TB burden in South Kivu Province and improve early detection efforts, contributing to achieving the Sustainable Development Goal of ending the TB epidemic by 2035.

## MATERIALS AND METHODS

The Mwenga HZ in South Kivu Province, DRC, spans 3969 km^2^ with an estimated population of 164 593 in 2021. Its healthcare system comprises 1 general referral hospital (GRH), 1 hospital center, 18 health centers, 10 health posts, and private facilities, including 3 Tuberculosis Diagnosis and Treatment Centers (DTCs): Kasika Health Center, Kalambi Hospital Center, and Mwenga GRH [10].

A cross-sectional study was conducted from July 24 to 24 August 2021, covering 13 of 18 health areas; 5 were excluded due to security and accessibility issues. Mwenga HZ was randomly selected from 4 TB-affected zones in South Kivu. The study identified 187 bacteriologically confirmed TB index cases from June to December 2020. Nurses and community health workers (CHWs) conducted index household and neighboring household awareness campaigns, investigating TB-symptomatic individuals.

The study included households with index cases of TB identified within the preceding 12 months. These households were required to have TB treatment records from one of the designated DTCs. Neighboring households were also included in the study to provide a broader perspective on TB transmission within the community. Furthermore, individuals exhibiting TB symptoms who consented to participate were eligible for inclusion.

Exclusion criteria ensured the study's focus and validity. Households located in inaccessible areas were excluded due to logistical constraints. Any subject undergoing treatment at the time of data collection; any index case identified in the management register, or any household member currently receiving antituberculosis therapy; and any subject who failed to provide 2 sputum samples. Additionally, participants who were unable to provide sputum samples or who had not resided with the index case before their diagnosis were not included in the study.

The dependent variables in the study were TB status, categorized as positive or negative. Independent variables included demographic and clinical factors such as age, sex, occupation, household size and type (defined as either an index case household or a neighboring household), duration of living together with the index case, previous TB treatment, clinical symptoms (eg, cough, fever, sweating, and lymphadenopathy), Bacille Calmette-Guerin (BCG) vaccination status, and awareness of TB and human immunodeficiency virus (HIV). These variables were carefully analyzed to identify potential risk factors and patterns associated with TB transmission within the community.

### Procedures

Nurses and CHWs raised awareness about TB and conducted household surveys using a structured, pretested questionnaire. TB symptoms were assessed, and 2 sputum samples were collected over 2 days. Samples were analyzed at Mwenga GRH using Ziehl-Neelsen staining, a conventional light microscopy technique specifically used for the detection of acid-fast bacilli to confirm active TB, with molecular (Xpert MTB/Rif [Xpert; Cepheid, Sunnyvale, CA]) testing conducted at the Provincial TB Laboratory. TB-positive cases were referred for treatment. All smear-positive TB patients received pretest counseling, and those who consented underwent HIV screening according to the National AIDS Control Program guidelines. Testing followed a serial algorithm using Alere Determine™ HIV-1/2 for initial screening, followed by Uni-Gold™ HIV for confirmation of reactive results.

### Data Analysis

Data were managed in Microsoft Excel and analyzed in R (Version 4.3.3; R Core Team, 2024). Quantitative variables were described using means or medians, compared using Student's *t*-test or Mann–Whitney–Wilcoxon test for mean and median, respectively. Qualitative variables were analyzed with Pearson chi-square or Fisher's exact test. Adjusted odds ratios (AOR) were calculated by logistic regression using the backward elimination model to assess associations between TB status and risk factors. A significance level of 5% was applied.

## RESULTS

A total of 10 141 individuals across 1122 households received health education on TB. Among them, 2121 (20.9%) were identified as presumed active pulmonary TB cases. Of these, 1707 (80.5%) participated in the study, while 414 (19.5%) did not. Reasons for nonparticipation included decline to participate (*n* = 197), children were unable to provide sputum samples (*n* = 97), poor sample quality (*n* = 62), and incomplete sampling where only 1 of the 2 required specimens was provided (*n* = 58).

Stratification of the 1707 participating presumed cases revealed that 493 (28.9%) resided in index case households, yielding a Ziehl-Neelsen positivity rate of 13.2% (*n* = 65). The remaining 1214 (71.1%) participants belonged to neighboring households, with a comparable Ziehl-Neelsen positivity rate of 12.7% (*n* = 154). Furthermore, despite diagnostic constraints, in the absence of radiological imaging facilities in this resource-limited setting, 31 children under 5 years of age were clinically diagnosed with 24 pulmonary and 7 extrapulmonary TB. In accordance with WHO diagnostic algorithms for paucibacillary childhood TB, these diagnoses were established based on a validated clinical scoring system combined with a thorough history of contact with a confirmed index case, highlighting the burden of the disease in vulnerable populations.

A total of 250 TB cases of pulmonary and extrapulmonary TB were confirmed during the study, yielding a prevalence of 14.66%. Among these, 219 cases were smear-positive, and 31 cases were clinically diagnosed. All confirmed active TB cases underwent HIV testing, with no positive results reported. Among the 219 smear-positive cases, Xpert MTB/RIF testing identified 4 cases of rifampicin-resistant TB. Of these cases, 2 were from index case households and 2 were from neighboring households. Notably, 3 of these cases were retreatment cases, while 1 was a new TB patient with no prior treatment history.

The analysis of data tables revealed significant differences between index case households and neighboring households in several aspects. Table 1 highlights statistically significant differences in the proportion of prior TB cases (*P* < .001) and the age distribution of presumptive cases (*P* = .049). Table 2 indicates significant differences in TB history within households (*P* < .001), the proportion of individuals without BCG vaccination (*P* < .001), in the prevalence of symptoms such as cough (*P* < .001), anorexia (*P* < .001), and night sweats (*P* < .001) between the 2 household types. However, no statistically significant difference was detected between the positive TB test results within index case households versus neighboring households (*P* = .84).

**Table 1.** General Characteristics of the Population by Type of Household

Variables	Index Case Households		Index Case Neighboring Households		*P*
	*n* = 493	Median (Min, Max) Mean (±SD) %	*n* = 1214	Median (Min, Max) Mean (±SD) %	
Age	493	38.39 (3–89)^a^	1214	36.96 (3–89)^a^	.22^e^
Household size	493	6.04 (2.50)^b^	1214	5.60 (2.51)^b^	.26^c^
Sex of presumed					.831^d^
Female	264	53.5	657	54.1	
Male	229	46.5	557	45.9	
Presumed status					<.001^d^
New patient	404	81.9	1193	98. 3	
TB retreatement	89	18.1	21	1.7	
Age group					.049^d^
<15 y	93	18.9	285	23.4	
15–64 y	342	69,4	789	63.3	
≥65 y	58	11.7	156	13.3	
Number of people in the households					.36^d^
≤5 persons	276	56.0	707	58.2	
>5 persons	217	44.0	507	41.8	
Profession of presumed					.37^d^
Farmer	315	63.9	718	59.1	
Miner	8	1.6	19	1.6	
Trader	14	2.8	49	4.0	
No professions	55	11.2	141	11.6	
Other professsions	101	20.5	287	23.7	

^a^Median (min-max).

^b^Mean ± SD.

^c^Student test.

^d^Pearson's chi-square test.

^e^Mann–Whitney.

**Table 2.** Distribution of Subjects According to Their Background and to Their Clinical Characteristics based on Household Type

Variable	Household of Index Cases		Household of Neighboring Cases		*P V*alue
	*N* = 493	%	*n* = 1214	%	
Antecedent of TB					<.001^a^
Yes	137	27.79	138	11.37	
No	356	72.21	1076	88.63	
Vaccinated with BCG					<.001^a^
Yes	167	33.9	258	21.2	
No	326	66.1	956	78.8	
Presumed TB receiving health education					.63^a^
Yes	279	56.59	704	57.99	
No	214	43.41	510	42.01	
Ziehl test result					.84^a^
Positive	65	13.18	154	12.69	
Negative	428	86.82	1060	87.31	
Cough					<.001^a^
Yes	453	91.89	976	80.40	
No	40	8.11	238	19.60	
Anorexia					<.001^a^
Yes	285	57.80	506	41.70	
No	208	42.20	708	58.30	
Tuberculous lymphadenopathy					.82^a^
Yes	10	2.03	21	1.73	
No	483	97.97	1193	98.27	
Fever					.31^a^
Yes	283	57.40	731	60.20	
No	210	42.60	483	39.80	
Night sweats					<.001^a^
Yes	289	58.60	586	40.30	
No	204	41.40	628	59.70	

Antecedent of TB: relapse, failure, and return after lost to follow-up.

^a^Pearson's chi-square test.

The adjusted analysis presented in Table 3 identified cough as a significant predictor of active TB, with an AOR of 1.89 (95% CI: 1.3–3.11, *P* = .008). Night sweats were also significantly associated with TB, with an AOR of 1.50 (95% CI: 1.12–2.00, *P* = .034). Additionally, age emerged as a significant factor, with an AOR of 1.01 (95% CI: 1.001–1.02, *P* = .018). These findings underscore the importance of targeted interventions to address risk factors and enhance TB control efforts in affected communities.

**Table 3.** Factors Associated With Tuberculosis

Variable	OR (95% CI)	*P V*alue	Adjusted OR (95% CI)	*P V*alue
Household of index cases versus neighboring cases (*n* = 1707)				
Index household	1.045 (.76–1.43)	.81	1.01 (.68–1.49)	.96
Neighboring	1		1	
Cough (*n* = 1707)				
Yes	1.86 (1.17–2.78)	.006	1.89 (1.2–3.11)	.008
No	1	-	1	-
Night sweats (*n* = 1707)				
Present	1.41 (1.06–1.87)	.02	1.50 (1.1246–2.00)	.006
Absent	1		1	
Age (*n* = 1707)				
<15 y	1		1	
15–64 y	1.12 (.77–1.62)	–	1.15 (.79–1.67)	.46
≥65 y	2.13 (1.34–3.37)	–	2.13 (1.34–3.37)	.001

Logistic regression reporting odds ratio (OR) and AOR (95% CI). The multivariable model adjusted a priori for cough, age group, and night sweats. Household of index cases versus neighboring cases as defined in Table 2.

## DISCUSSION

Although this is the first cross-sectional study in South Kivu Province to evaluate TB prevalence and risk factors among both index and neighboring households, its short 2-month duration and reliance on sputum smear microscopy—owing to the unavailability of culture and molecular diagnostics—may have led to an underestimation of the true number of cases due to the lower sensitivity of this method.

A total of 187 TB index cases were identified 1 year after their diagnosis. Of the 2121 presumptive TB cases in these households, 1707 (80.5%) participated in the study. Among these, 250 TB cases (14.65%) were confirmed across all forms, including 219 smear-positive confirmed cases (12.83%). The prevalence of TB did not show a statistically significant difference (*P* = .84) between index case households (13.2%) and neighboring households (12.7%).

In this study, 80.5% of eligible participants underwent TB screening. Similar studies in high TB prevalence settings have reported varying levels of participation. For instance, 85% participation was achieved in South India [14], comparable to our results. However, lower participation rates were observed in Bangkok, Thailand (52%) [15], Uganda (50%) [16], and rural Cape Town, South Africa (39%) [17]. The relatively high participation in our study can be attributed to the involvement of CHWs and nurses familiar with the targeted communities, as well as the convenience of sputum collection being conducted at participants’ homes.

The study identified 1 new TB case for every 8 contacts screened, a higher yield compared to 15 contacts per case in a South African study [18]. The observed prevalence among index case contacts (13.2%) is significantly higher than the national average of 8.1% reported by the DRC National TB Program (PNLT) [19] and the provincial average of 6.5% in South Kivu [9]. In Mwenga HZ, only 3.8% of the 339 TB cases notified in 2021 were identified through contact investigation [10]. These findings highlight that routine contact investigation remains suboptimally implemented. The high yield of cases identified in this study demonstrates that active case-finding targeting close contacts can uncover a significant burden of disease that traditional passive surveillance fails to detect, even when utilizing a diagnostic tool with limited sensitivity.

Studies in Uganda [20] and South Africa [21] reported varying TB prevalence among contacts, ranging from 7.8% to 10.1%. Prevalence rates comparable to our findings were observed in Myanmar (13.8%) [22], while lower rates were reported in Kenya (10%) [23], Shanghai, China (5.5%) [24], Burkina Faso (8.1%) [8], Uganda (5.3%) [25], southern Ethiopia (5.1%) [26], and Tanzania (6.4%) [27]. A systematic review of 95 studies in low-and middle-income countries reported family contact TB prevalence ranging from 2.1% to 10.1% [21]. Higher prevalence rates were found in Pakistan (27.7%) [28], which is one of the most TB-endemic countries globally, surpassing the DRC.

Few studies have focused on TB screening in neighboring households of index cases. Our findings showed no statistically significant difference (*P* = .84) between TB prevalence in index households (13.2%) and neighboring households (12.7%). Similar results were reported in Uganda, where TB prevalence among first-degree relatives not living with the index case was comparable to that among household contacts [29]. However, a study by Gashu Z et al in Ethiopia found a higher likelihood of TB among neighbors (AOR: 1.35, 95% CI: 1.02–1.78) and workplace contacts (AOR: 3.95, 95% CI: 2.21–7.03) compared to household contacts [30].

Our results align with studies that highlight the interconnectedness of rural communities, where social interactions and regular visits among neighbors and family members facilitate the transmission of communicable diseases, including TB. This makes it challenging to distinguish true contacts from casual interactions. A study in Ho Chi Minh City, Vietnam, suggested that neighborhood-level screening within a 50-m radius of index cases, particularly for retreatment or migrant cases, could be an effective strategy to curb TB transmission [31].

Rather than merely confirming well-known symptoms, the persistence of cough, night sweats, and age as significant factors across both household cohorts underscores their operational value. In resource-limited settings lacking radiological infrastructure, this specific clinical profile serves as a robust, high-yield triaging tool to guide active case-finding and optimize the use of suboptimal diagnostic methods like smear microscopy.

Studies in South Africa [18], Tanzania [32], and Uganda [20] have similarly identified cough, older age, and close contact (eg, sharing a room) as significant TB risk factors. A study in Peru linked TB diagnosis with older age (OR = 5.5, 95% CI: 1.2–22.8) and female sex [33], while family history and extended family living arrangements were significant in Indonesia [34].

The prevalence of rifampicin-resistant TB (1.8%) in our study was lower than the 11.1% reported in South Kivu by Bulabula et al [35]. This discrepancy could be due to the lower diagnostic sensitivity of Ziehl-Nelsen microscopy compared to GeneXpert, as well as the limited scope of our study to Mwenga HZ over a short period.

Finally, no TB-HIV co-infection was observed among the 219 TB cases tested for HIV. This aligns with Mwenga HZ's 2021 data, which reported only 2 HIV-positive cases among 339 TB patients [9], indicating a low HIV prevalence in the region.

## CONCLUSION

This study demonstrates a similar risk of contracting TB in both index case and neighboring households, highlighting the need for contact tracing in both environments. Coughing was identified as a critical symptom justifying community-based screening, while age over 65 years represents a significant risk factor for disease development. Although our laboratory methodology relied on smear microscopy which carries a lower sensitivity, the household prevalence of 13.2% (approximately twice the provincial average) does not imply a superior technical performance of our tool. Rather, it underscores a substantial community under-detection in the Mwenga HZ, driven by the public health system's reliance on passive case-finding, which fails to proactively identify symptomatic contacts who have not yet sought healthcare. To address these gaps, this study proposes a practical field-approach designed to enhance early detection and curtail transmission. These findings offer actionable insights for TB control programs in the DRC and similar high-burden settings. Future research should expand the screening radius beyond 50 m to better define the operational limits of this contact-tracing strategy.
